# The Yeast F-Box Protein Met30 Regulates Proline Utilization Independently of Transceptor Can1 Under Nutrient-Rich Conditions

**DOI:** 10.3390/microorganisms12122510

**Published:** 2024-12-05

**Authors:** Akira Nishimura, Ryoya Tanahashi, Hiroshi Takagi

**Affiliations:** 1 Institute for Research Initiatives, Nara Institute of Science and Technology, 8916-5 Takayama-cho, Ikoma 630-0192, Nara, Japan; tanahashi.ryoya.ti3@bs.naist.jp; 2 Department of Food Science and Technology, University of California Davis, One Shields Ave., Davis, CA 95616, USA

**Keywords:** amino acids, fermentations, proline utilization, *Saccharomyces cerevisiae*, SCF ubiquitin ligase

## Abstract

Proline is the most abundant amino acid in wine and beer, largely due to the limited utilization of proline by the yeast *Saccharomyces cerevisiae* during fermentation. Previous studies have shown that the arginine transporter Can1 plays a role in regulating proline utilization by acting as a transceptor, combining the functions of both a transporter and a receptor for basic amino acids. However, the *CAN1*-disrupted strains have exhibited the inhibition of proline utilization under nutrient-rich conditions, indicating that additional factors beyond basic amino acids contribute to the inhibition of proline utilization. Here, we used the parent strain with the *CAN1* deletion to derive mutants that can utilize proline even under nutrient-rich conditions. A genomic analysis revealed a mutation in the *MET30* gene, which encodes an F-box subunit of the SCF ubiquitin ligase complex, that causes reduced Met30 function. Importantly, we found that Met30 and Can1 independently regulate proline utilization. Our screening showed that the Met30-dependent inhibition of proline utilization occurs when ammonium ions, methionine or cysteine, and another amino acid (especially threonine or isoleucine) are present simultaneously. The present data offer new insights into the regulation of proline metabolism.

## 1. Introduction

For the production of alcoholic beverages, selecting yeast strains with the appropriate characteristics is crucial for controlling the aroma and flavor while ensuring effective ethanol production. The metabolic properties of yeast cells significantly influence the quality of these beverages. Grape musts used in wine fermentation have adequate sugar levels to support optimal yeast growth. However, the nitrogen content in grape musts can vary widely, typically ranging from 60 to 2400 mg/L. Nitrogen availability is a key factor in wine fermentation, affecting both the rate and duration of the fermentation process [1].

Grape musts are rich in proline, an amino acid that can serve as a nitrogen source [2,3,4]. However, during fermentation, the wine yeast *Saccharomyces cerevisiae* (*S. cerevisiae*) cannot utilize proline, resulting in nitrogen deficiency and proline accumulation in the finished wine [5,6]. Additives such as diammonium phosphate are commonly introduced during fermentation to address the nitrogen deficiency. These additives can alter yeast metabolisms, potentially changing the taste and aroma of wine. Additionally, such interventions may promote the formation of ethyl carbamate, a potential carcinogen [7]. Excessive proline levels in wine can also affect the sweetness and acidity [8,9]. Therefore, developing yeast strains capable of utilizing proline presents a promising approach to enhance fermentation efficiency and improve wine quality.

Previous genetic research has shown that ammonium ions, representing up to 10% of the total assimilable nitrogen in grape musts, inhibit proline metabolism through nitrogen catabolite repression (NCR) during fermentation [10]. However, our recent studies have demonstrated that basic amino acids, except for histidine, are more effective inhibitors of proline utilization than ammonium ions and act independently of the NCR system [11]. Basic amino acids hinder proline metabolism by deactivating the proline-specific transporter Put4. According to our earlier model, basic amino acids promote the ubiquitination of Put4 through the Nedd4 family ubiquitin ligase Rsp5 and the α-arrestin protein Art3, leading to the endocytosis of Put4 [12]. Recent findings have also revealed that the arginine transporter Can1 impacts proline metabolism independently of its arginine uptake activity [13]. Can1 activates protein kinase A signaling pathways in response to extracellular basic amino acids without increasing cAMP levels [14]. This suggests that Can1 functions as a “transceptor”, acting both as a transporter and a receptor for basic amino acids. These insights suggest a mechanism by which Can1 mediates the sensing of basic amino acids to inhibit proline utilization. However, the effect of deleting the *CAN1* gene on proline metabolism during wine fermentation has yet to be investigated

In this study, we first screened for mutant strains with enhanced proline utilization under nutrient-rich conditions, and then conducted a whole-genome sequencing analysis on the obtained strains. As a result, we found that a *MET30* mutation (Asp361Gly) facilitates proline utilization. We also determined that Met30, a component of the SCF ubiquitin ligase complex, is involved in proline utilization in the presence of ammonium ions, methionine or cysteine, and any amino acid except valine, tryptophan, or leucine. A gene expression analysis revealed the increased expression of Met4-regulated genes in the Met30 mutant, suggesting the constitutive activation of Met4 due to the reduced Met30 function. Based on our findings, we propose a regulatory mechanism whereby various nitrogen sources in the environment mediate proline utilization through Met30. This study provides insights into the intricate regulation of proline metabolism with implications for wine fermentation and yeast breeding.

## 2. Materials and Methods

### 2.1. Culture Medium

The media used in this study included a synthetic proline-containing medium (SD-N+Pro), a white grape medium (WG; Brix value: 18.3%), a yeast extract–peptone–dextrose medium (YPD), and a synthetic complete medium (SC). The SD-N+Pro comprised 2% glucose, 0.67% yeast nitrogen base without amino acids and ammonium sulfate (Difco Laboratories, Detroit, MI, USA), and 0.1% proline. The WG contained 2% of a commercially available white grape must (Alps, Nagano, Japan). The YPD contained 1% yeast extract (Difco Laboratories), 2% peptone (Difco Laboratories), and 2% glucose. The SC was prepared with 2% glucose, 0.67% yeast nitrogen base lacking ammonium sulfate and amino acids (Difco Laboratories), and a supplement of 0.002% adenine, 0.04% leucine, 0.0008% p-aminobenzoic acid, 0.008% uracil, and a range of amino acids as follows: arginine, aspartic acid, glutamine, glycine, inositol, methionine, phenylalanine, serine, tryptophan, alanine, asparagine, cysteine, glutamic acid, histidine, isoleucine, lysine, proline, threonine, tyrosine, and valine. The pH of all the media was adjusted to 6.5. A total of 2% agar (Nacalai Tesque, Kyoto, Japan) was added when required.

### 2.2. Strains

Table S1 lists the eight yeast strains used in this study, including the wild type (WT) and various mutants: *pro1*Δ*car2*Δ, *pro1*Δ*car2*Δ*can1*Δ, *pro1*Δ*car2*Δ*can1*Δ*met30*^D361G^, *pro1*Δ*car2*Δ*met30*^D361G^, *can1*Δ, *met30*^D361G^, and *can1*Δ*met30*^D361G^. The strains *pro1*Δ*car2*Δ, *pro1*Δ*car2*Δ*can1*Δ, and *can1*Δ were generated in previous studies [13]. Table S2 provides the details of the primers used.

To generate strains expressing the *met30*^D361G^ variant chromosomally, a CRISPR-Cas9 system was employed. A gRNA and Cas9 expression plasmid (pCas9-MET30) targeting the *MET30* locus were constructed using the QuikChange method, with primers gRNA-MET30 Fw and gRNA-MET30 Rv and the plasmid pCas9 (obtained from AddGene). The transformation into yeast cells was performed using the LiAc/SS carrier DNA/PEG method [15], with 1 μg of pCas9-MET30 and 1 nmol of double-stranded oligonucleotides. These oligonucleotides, produced by mixing equimolar amounts of MET30 A1082G dsDNA Fw and Rv, were subjected to a temperature cycle of 100 °C for 5 min and gradual cooling to 25 °C at 0.1 °C per second. The transformants were plated on the SD-N+Pro medium containing 350 μg/mL G418, and the desired mutation was confirmed by DNA sequencing.

### 2.3. Spot Test

The yeast cells were diluted with water to an optical density at 600 nm (OD_600_) of 1.0. Ten-fold serial dilutions were prepared, and aliquots were spotted onto the indicated media as described in the figure legends. The plates were incubated at 30 °C for 2–3 days.

### 2.4. Isolation of Proline-Utilizing Mutants Under Nutrient-Rich Media

The strain *pro1*Δ*car2*Δ*can1*Δ was grown to the stationary phase in the SD-N+Pro medium at 30 °C with shaking. After two washes with sterile water, approximately 10^9^ cells were plated on the YPD medium. Two colonies, Mutant-1 and Mutant-2, were selected after 3 days of incubation at 30 °C.

### 2.5. Whole-Genome Sequencing

Mutant-1 and Mutant-2 were cultured in the YPD medium, and their genomic DNA was extracted using a Dr. GenTLE (from yeast) high-recovery kit (Takara Bio, Shiga, Japan). The libraries were prepared with a NEB Next Ultra DNA Library Prep Kit (New England Biolabs, Ipswich, MA, USA), and the paired-end 150 bp reads were sequenced using the Illumina NovaSeq 6000 platform (Illumina, San Diego, CA, USA) via a commercial DNA sequencing service (Rhelixa, Tokyo, Japan).

### 2.6. Measurement of Residual Amino Acids

The yeast strains precultured in the SD-N+Pro medium were inoculated into the WG medium at an initial OD_600_ of 1.5. The cultures were incubated statically at 25 °C for 24, 48, and 72 h. The supernatants were collected by centrifugation, and the residual amino acid levels were measured using an amino acid analyzer (JLC-500/V; JEOL, Tokyo, Japan).

### 2.7. RNAseq Analysis

The yeast strains were precultured to the stationary phase in the SD-N+Pro medium. The cells were washed twice with distilled water and resuspended in the WG medium at an OD_600_ of 1.0. After a 3 h incubation, the cells were disrupted using a multi-beads shocker (MB601U; Yasui Kikai, Osaka, Japan) with 0.5 mm glass beads. The RNA was extracted with a NucleoSpin RNA Plus kit (Takara Bio) following the manufacturer’s instructions. The RNA sequencing was performed by a commercial service (Rhelixa).

### 2.8. Quantitative PCR Analysis of SUL1

The WT cells precultured in the SD-N+Pro medium were inoculated into the SD-N+Pro supplemented with various combinations of methionine, ammonium ions, threonine, and valine at an initial OD_600_ of 1.0. After a 3 h incubation, the cells were disrupted as described above. The total RNA was extracted using a NucleoSpin RNA Plus kit, and the cDNA was synthesized with a PrimeScript RT reagent kit (Takara Bio). A quantitative PCR was performed on a QuantStudio3 system (Thermo Fisher Scientific, Waltham, MA, USA) using SsoAdvanced Universal SYBR Green Supermix (Bio-Rad Laboratories, Hercules, CA, USA). The primers for *SUL1* and *ACT1* (Table S2) were used with 96.2% and 97.8% efficiencies, respectively. The relative mRNA levels were calculated using the 2^−ΔΔCt^ method, normalized to the *ACT1* expression.

### 2.9. Statistical Analysis

The data are presented as the means ± standard deviation (SD). Statistical significance was assessed using a one-way or two-way analysis of variance (ANOVA) followed by Tukey’s test, conducted with Prism 7 software version 7.05 (GraphPad, San Diego, CA, USA). The results with *p* < 0.05 were considered statistically significant.

## 3. Results and Discussion

**Figure 1:**
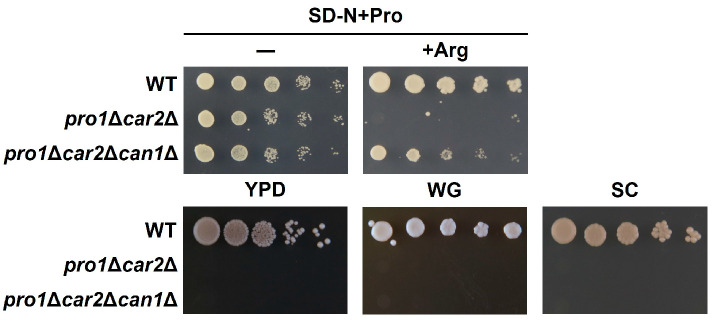
*CAN1*-dependent inhibition of proline utilization. WT, *pro1*Δ*car2*Δ, and *pro1*Δ*car2*Δ*can1*Δ strains were precultured in SD-N+Pro and spotted onto SD-N+Pro (−), SD-N+Pro with arginine (+Arg), YPD, WG, and SC mediums.

Based on a method previously developed by our group, proline utilization was evaluated by using the proline auxotrophic strain *pro1*Δ*car2*Δ and media containing proline as the sole nitrogen source (SD-N+Pro) [11]. *S. cerevisiae* possesses two biosynthetic pathways for proline: the glutamate pathway and the arginine pathway. The key enzymes involved in these pathways are *γ*-glutamyl kinase, encoded by the *PRO1* gene [16], and ornithine aminotransferase, encoded by the *CAR2* gene [17]. Consequently, the *pro1*Δ*car2*Δ strain exhibits a defect in proline synthesis, leading to proline auxotrophy. As shown in Figure 1, the wild-type (WT) cells grow normally on the medium containing proline as the sole nitrogen source regardless of arginine addition. On the other hand, the *pro1*Δ*car2*Δ cells grow on the medium containing only proline, but do not grow on the medium containing arginine (+Arg). The *CAN1* deletion cancels the growth inhibition of the *pro1*Δ*car2* cells on the medium with arginine. These data agree with a previous report that found that Can1 is involved in the basic amino acids-inducible inhibition of proline utilization [13]. Next, we determined the proline utilization of the cells in three different nutrient-rich media: yeast extract–peptone–dextrose (YPD) medium, white grape juice (WG) medium, and complete synthetic (SC) medium (Figure 1). The results indicate that the WT cells can grow in all the media, whereas not only the *pro1*Δ*car2*Δ cells, but also the *pro1*Δ*car2*Δ*can1*Δ cells cannot grow in the nutrient-rich media. Thus, nutrient-rich media may contain unidentified inhibitory factors of proline utilization other than basic amino acids.

**Figure 2:**
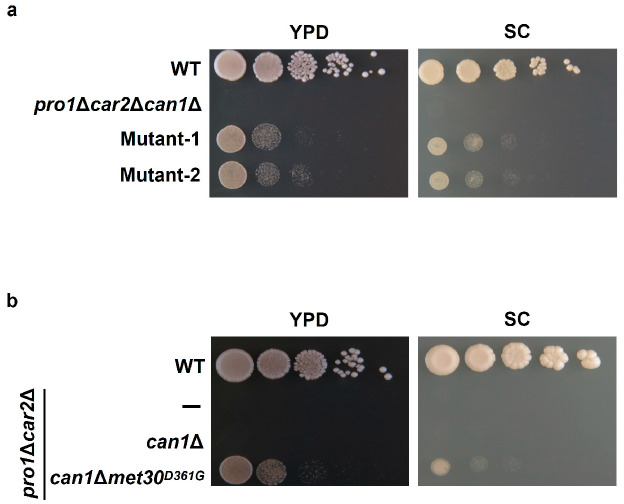
Screening of mutants that utilize proline under nutrient-rich conditions. (**a**) Isolation of spontaneous proline auxotrophic mutants that can grow under nutrient-rich conditions. WT, *pro1*Δ*car2*Δ*can1*Δ, and selected mutant strains (designated Mutant-1 and Mutant-2) were spotted onto YPD and SC mediums. (**b**) Identification of gene involved in inhibition of proline utilization. WT, *pro1*Δ*car2*Δ, *pro1*Δ*car2*Δ*can1*Δ, and *pro1*Δ*car2*Δ*can1*Δ*met30^D361G^* strains were spotted onto YPD and SC mediums.

**Figure 3:**
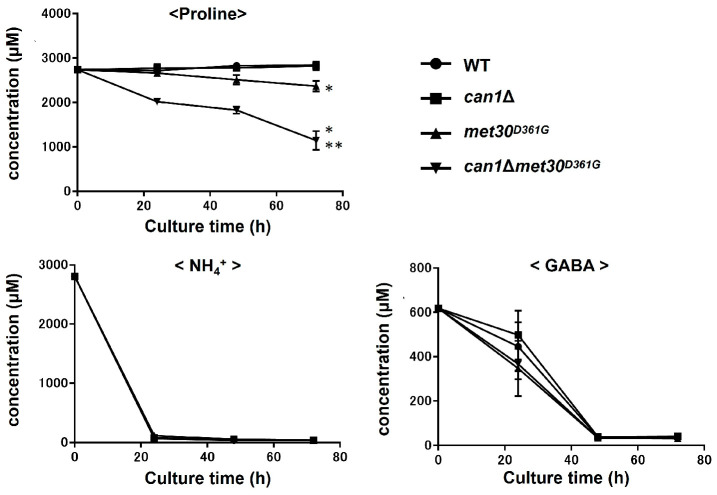
Proline consumption of a prototroph yeast strain with *met30^D361G^* mutation in a wine fermentation model. The WT, *can1*Δ, *met30^D361G^*, and *can1*Δ*met30^D361G^* strains were inoculated into the WG medium. The proline amount was measured every 24 h under static conditions. The relative proline content at 0 h was taken as 100%. The data are presented as the means ± SD (*n* = 3), and statistical significance was determined by a two-way ANOVA with Tukey’s test. * *p* < 0.05 vs. WT; ** *p* < 0.05 vs. *met30^D361G^*.

**Figure 4:**
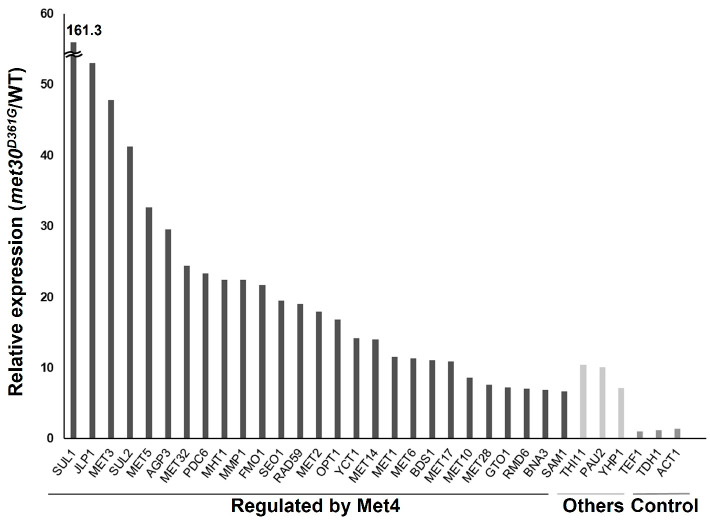
Comprehensive gene expression analysis of the *met30* mutant. An RNAseq analysis of the WT and *met30^D361G^* strains was conducted in the WG medium. The top 30 genes with higher expression levels in the *met30^D361G^* strain compared to the WT strain are depicted graphically. The presented genes are classified as either Met4-activated genes (Regulated by Met4) or others (Others). The genes commonly employed as internal controls (*TEF1*, *TDH1*, and *ACT1*) served as the control group (Control).

To elucidate the inhibitory mechanisms of proline utilization other than the Can1 pathway, we screened the mutants that can utilize proline even in nutrient-rich media. By using a screening with the strain *pro1*Δ*car2*Δ*can1*Δ (approximately 10^9^ cells) and YPD medium, we obtained two spontaneous mutants (Mutant-1 and Mutant-2) that could grow on the YPD medium (Figure 2a). Mutant-1 and Mutant-2 could also grow on the SC medium (Figure 2a). A whole-genome sequencing analysis revealed that both Mutant-1 and Mutant-2 have a nucleotide G at position 1082 on the locus of *MET30*, encoding an F-box protein as part of the ubiquitin ligase complex. In contrast, the *pro1*Δ*car2*Δ*can1*Δ and laboratory yeast strains with publicly available genomic information (*Saccharomyces* Genome Database: https://www.yeastgenome.org [accessed on 22 May 2021]) have a nucleotide A at the same position. This mutation of A to G (1082 A>G) leads to the amino acid substitution of Asp to Gly at position 361 (D361G). This mutation involving an amino acid substitution was only found in the *MET30* locus. We next introduced this mutation into the genome of strain *pro1*Δ*car2*Δ*can1*Δ using a CRISPR/Cas9 system to verify the effect of this mutation on proline utilization. As indicated in Figure 2b, the mutant strain *pro1*Δ*car2*Δ*can1*Δ*met30^D361G^* shows growth on the YPD and SC, while the parent strain *pro1*Δ*car2*Δ*can1* does not grow on any of the media. These results demonstrate that the combination of the missense mutation (1082 A>G) of *MET30* and a *CAN1* deletion cancel the inhibition of proline utilization by rich nutrients, indicating that Met30 is involved in the inhibition of proline utilization in nutrient-rich media. Met30 is an F-box protein that is one of the components of the Skp1/Cullin/F-box (SCF) ubiquitin ligase complex (Figure S1) [18]. F-box proteins are essential for the direct binding of the SCF ubiquitin ligase complex to substrates [19]. The main function of the SCF complex containing Met30 (SCF-Met30) is the regulation of sulfur metabolism. One of the representative substrates of SCF-Met30 is Met4, a transcription factor involved in sulfur metabolism [18]. Under normal conditions, Met4 is bound to Met30 and ubiquitinated by SCF-Met30, inhibiting its transcriptional activity. Conversely, when sulfur sources are depleted, SCF-Met30 dissociates, leading to the deubiquitination of Met4. In addition, the downstream genes of Met4 are rapidly expressed. Met4 controls almost all the sulfur-related genes, including sulfate transporters, cysteine, methionine, and glutathione synthesis genes. Met30 directly binds to the substrates by the tryptophan and aspaginate 40 (WD40) repeat domain within itself [20]. The mutation point (Asp residue at position 361) identified in this study is located within the WD40 repeat domain and is highly conserved in fungi (Figure S2). We next constructed the prototroph yeast strains *met30^D361G^* and *can1*Δ*met30^D361G^* to determine the level of proline consumption in a wine fermentation model (WG medium; static condition, 25 °C). Proline consumption was not observed even after 72 h of cultivation in strains WT and *can1*Δ, whereas strain *met30^D361G^* showed a slight consumption of proline (Figure 3). Strain *can1*Δ*met30^D361G^* significantly consumed proline from the initial stage of cultivation (approximately 50% after 72 h). The strains with the mutation in *MET30* (*met30^D361G^* and *can1*Δ*met30^D361G^*) appeared to have an increased consumption of γ-aminobutyric acid (GABA) compared to the strains without mutations (WT and *can1*Δ), although the difference was not statistically significant. For the ammonium ions, the consumption rate remained unchanged across all the strains. Yeast cells have the ability to assess the quality of nitrogen sources [21,22,23]. Usually, the presence of favorable nitrogen sources, like ammonium ions, inhibits the use of less favorable sources, such as proline and GABA, through a mechanism known as NCR. However, there were few significant differences observed in the consumption of GABA among the different strains. Our data indicate that Can1 and Met30 have a minimal impact on the NCR. We then conducted a comprehensive gene expression analysis by RNAseq for the WT and *met30^D361G^* strains to examine the impact of the *MET30* mutation. A comparison of their gene expression showed that among the top 30 genes with increased expression in strain *met30^D361G^*, 27 genes are regulated by Met4 (Figure 4) [24]. Beyond the Met4-regulated genes, *THI11* encodes a protein involved in the synthesis of the thiamine precursor [25]. *PAU2* encodes a protein of unknown function belonging to the seripauperin multigene family [26]. *YHP1* encodes a homeobox transcription factor that regulates gene expression late in the cell cycle [27]. There was no increase in the expression of the representative control genes (*TEF1*, *TDH1*, and *ACT1*). Under rich-nutrient conditions, Met4 is negatively regulated by SCF-Met30, which represses the expression of the genes controlled by Met4 [18]. Therefore, this mutation (1082 A>G, D361G) reduces the Met30 function, leading to constitutive activations of Met4. These data indicate that a disruption or reduction in the functions of both Can1 and Met30 facilitates proline utilization, even under conditions permissible for wine fermentation. Thus, we conclude that Met30 is one of the regulators of proline utilization. In addition to Met4, Met32, Cse4, and Atg9 have been identified as substrates for SCF-Met30 [24,28,29,30,31,32]. Met32, similar to Met4, plays a role in sulfur metabolism, while Cse4 is involved in chromosome segregation, and Atg9 is associated with autophagy. Therefore, Met30 regulates not only sulfur metabolism but also various other cellular functions. However, there has been no report showing that SCF-Met30 can ubiquitinate the proteins associated with proline metabolism. One hypothesis to account for the absence of such a finding is that there are unknown substrates of Met30. In general, the interaction between the F-box protein and its substrate is weak, making it difficult to identify the substrates using conventional methods, such as the two-hybrid system or immunoprecipitation. Recently, it was demonstrated that a temperature-sensitive mutation in *CDC34*, which encodes the catalytic subunit of the SCF complex, can be utilized in the two-hybrid method to inhibit ubiquitination [33]. This results in stronger binding between the F-box protein and its substrate, facilitating substrate identification. Therefore, we plan to use this system in the future to search for the unknown substrates of Met30.

**Figure 5:**
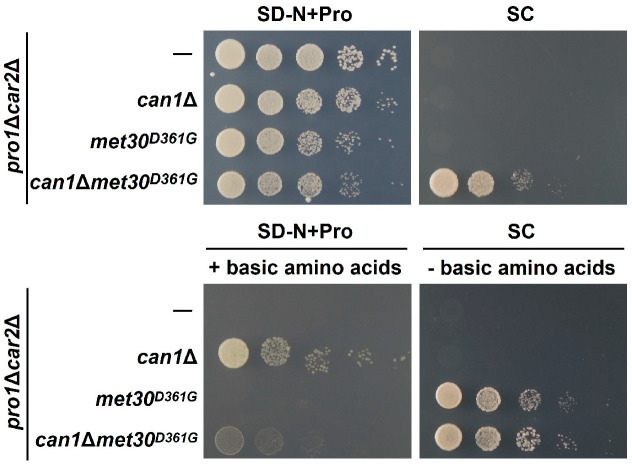
The *MET30*-dependent inhibition of proline utilization independent of the Can1 pathway. The *pro1*Δ*car2*Δ, *pro1*Δ*car2*Δ*can1*Δ, *pro1*Δ*car2*Δ*met30^D361G^*, and *pro1*Δ*car2*Δ*can1*Δ*met30^D361G^* strains were spotted onto the SD-N+Pro with/without basic amino acids and the SC medium with/without basic amino acids.

Based on our present findings, we hypothesize that Met30 and Can1 control proline utilization through independent pathways. To test this hypothesis, we evaluated the proline utilization in each strain in the SD-N+pro medium supplemented with arginine and lysine (SD-N+Pro+basic amino acids) and in the SC medium without arginine and lysine (SC-basic amino acids) (Figure 5). We constructed strain *pro1*Δ*car2*Δ*met30^D361G^* using a CRISPR/Cas9 system. Figure 5 shows that *pro1*Δ*car2*Δ*met30^D361G^*, like *pro1*Δ*car2*Δ*can1*Δ, does not grow on the SC medium. Notably, the growth of *pro1*Δ*car2*Δ*can1*Δ is observed on the SD-N+Pro+basic amino acids in a manner similar to that of *pro1*Δ*car2*Δ*can1*Δ*met30^D361G^*, while the growth of *pro1*Δ*car2*Δ*met30^D361G^* is completely inhibited. Conversely, the *pro1*Δ*car2*Δ*met30^D361G^* and *pro1*Δ*car2*Δ*can1*Δ*met30^D361^* strains exhibit clear growth on the SC-basic amino acids, but *pro1*Δ*car2*Δ*can1*Δ does not grow on this medium. These findings suggest the presence of two groups of proline utilization inhibitory factors under nutrient-rich conditions, one dependent on Can1-mediated basic amino acids (arginine and lysine), and the other dependent on unknown factors associated with Met30. These data also support our hypothesis that Met30 and Can1 control proline utilization independently.

**Figure 6:**
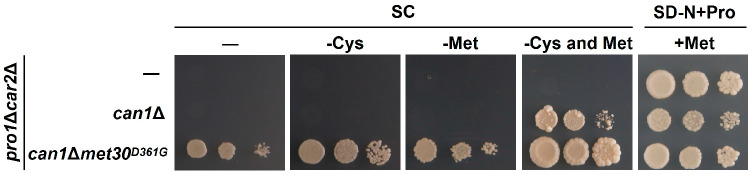
Sulfur-containing amino acids-dependent inhibition of proline utilization. The *pro1*Δ*car2*Δ, *pro1*Δ*car2*Δ*can1*Δ, and *pro1*Δ*car2*Δ*can1*Δ*met30^D361G^* strains were spotted onto the SC, SC without cysteine or/and methionine, and SD-N+Pro with methionine.

**Figure 7:**
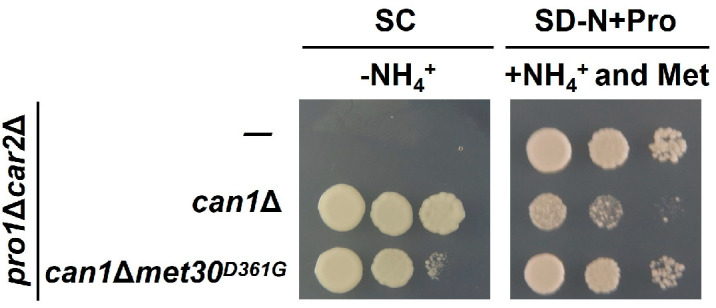
Ammonium ion-dependent inhibition of proline utilization. The *pro1*Δ*car2*Δ, *pro1*Δ*car2*Δ*can1*Δ, and *pro1*Δ*car2*Δ*can1*Δ*met30^D361G^* strains were spotted onto the SC without NH_4_^+^ and SD-N+Pro with NH_4_^+^+methionine.

**Figure 8:**
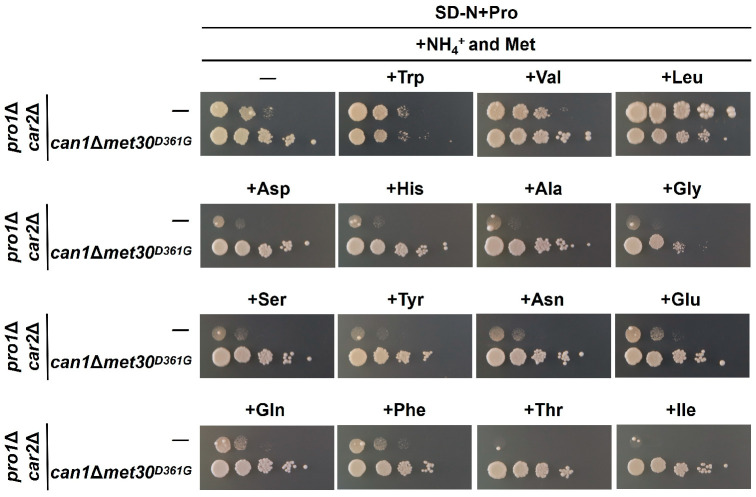
The identification of proline utilization inhibitors in the presence of ammonium ions and methionine. The *pro1*Δ*car2*Δ and *pro1*Δ*car2*Δ*can1*Δ*met30^D361G^* strains were spotted onto the SD-N+Pro with ammonium ions (NH_4_^+^) and methionine plus another amino acid.

Since the activity of Met30 is controlled by sulfur-containing amino acids (methionine and cysteine) [34], we evaluated proline utilization on the SC media lacking cysteine, methionine, or both (SC-Cys, SC-Met, and SC-Cys-Met). As shown in Figure 6, the growth of the *pro1*Δ*car2*Δ and *pro1*Δ*car2*Δ*can1*Δ strains is completely inhibited in the media excluding solely cysteine and solely methionine. In contrast, the growth of *pro1*Δ*car2*Δ*can1*Δ is observed in the medium excluding both cysteine and methionine. Strain *pro1*Δ*car2*Δ*can1*Δ*met30^D361G^* grows in all the media. These findings suggest that methionine and cysteine may serve as inhibitory factors of proline utilization. However, the addition of methionine (SD-N+Pro+Met) to the medium containing proline as the sole nitrogen source does not inhibit the growth of *pro1*Δ*car2*Δ and *pro1*Δ*car2*Δ*can1*Δ (Figure 6). Hence, methionine and cysteine may act in concert with other inhibitors of proline utilization other than basic amino acids. Next, we prepared an SC medium lacking ammonium ions (SC-NH_4_^+^) to investigate the inhibitory effect of ammonium ions. Figure 7 indicates that *pro1*Δ*car2*Δ*can1*Δ grow on the SC-NH_4_^+^, but *pro1*Δ*car2*Δ does not. In contrast, *pro1*Δ*car2*Δ grows even on the medium supplemented with both ammonium ions and methionine (SD-N+Pro+NH_4_^+^+Met). Thus, ammonium ions clearly act as inhibitors of proline utilization, but ammonium ions may act with other factors rather than alone. We then added these amino acids one by one to the medium that was supplemented with both ammonium ions. The growth of *pro1*Δ*car2*Δ in this medium was comparable to the growth of *pro1*Δ*car2*Δ in the control medium when tryptophan, valine, or leucine was added (Figure 8). More surprisingly, the growth of *pro1*Δ*car2*Δ was suppressed on the media supplemented with other amino acids (aspartic acid, histidine, alanine, glycine, serine, tyrosine, asparagine, glutamic acid, glutamine, phenylalanine, threonine, or isoleucine) (Figure 8). Notably, the addition of threonine and isoleucine significantly inhibited cellular growth. The strain *pro1*Δ*car2*Δ*can1*Δ*met30*^D361G^ showed normal growth in all the media (Figure 8). Thus, these data suggest that the simultaneous presence of three factors—(1) ammonium ions, (2) methionine or cysteine, and (3) any amino acids except valine, tryptophan, or leucine—strongly inhibits proline utilization in a Met30-dependent manner.

**Figure 9:**
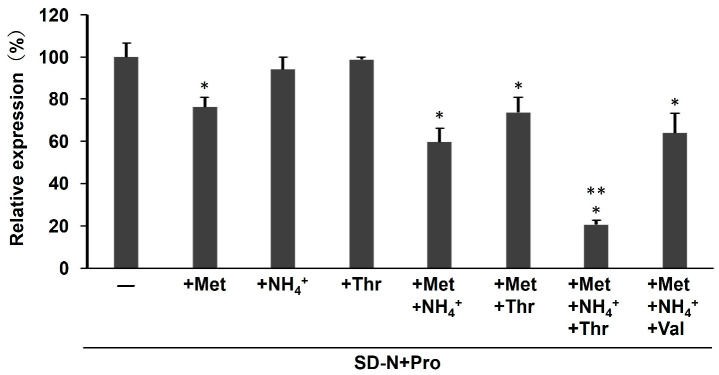
*SUL1* expression under the inhibitory condition of proline utilization. The *SUL1* expression was determined during growth in the SD-N+Pro with various combinations of methionine, ammonium ions (NH_4_^+^), threonine, and valine. The data are presented as the means ± SD (*n* = 3), and statistical significance was determined by a one-way ANOVA with Tukey’s test. * *p* < 0.05 vs. SD-N+Pro; ** *p* < 0.05 vs. SD-N+Pro+Met.

Finally, we examined the impact of three factors—ammonium ions, methionine, and threonine—on the activation of Met30 by determining the expression of the *wine* gene, which is regulated by Met4. Figure 9 shows that the addition of only methionine to the medium containing proline slightly reduces the expression of *SUL1* compared with the addition of ammonium ions and threonine, indicating that methionine activates Met30, as previously reported. However, the difference in the *SUL1* expression between the two combinations is not significant (Figure 9). More importantly, the simultaneous addition of ammonium ions, methionine, and threonine, a condition under which proline utilization inhibition occurs, dramatically decreases the expression of *SUL1* (Figure 9). The simultaneous addition of ammonium ions, methionine, and valine, a condition under which the inhibition of proline utilization does not occur, has similar results to the addition of methionine alone. Hence, the present results indicate that not only methionine, but also ammonium ions and threonine, are intricately involved in the regulation of the SCF-Met30 pathway. Previous studies have proposed that sulfur-containing amino acids, such as methionine and cysteine, activate the SCF-Met30 complex, leading to the ubiquitination of substrates such as Met4 [34]. However, the precise mechanism of SCF-Met30 action remains largely unclear. The present data suggest that SCF-Met30 is activated primarily by the simultaneous presence of other nitrogen sources, such as ammonium ions and threonine, rather than by sulfur-containing amino acids alone. The inhibitory effects of each amino acid on proline utilization vary widely, with no apparent consistency regarding the side chains or structures. Hence, the nitrogen catabolite repression system, which has been suspected as contributing to the inhibition of proline utilization, may not be involved in the Met30 mechanism. Yeast is well known to possess a TORC1 system for sensing nitrogen sources [35,36]. TORC1 serves as an intracellular amino acid sensor complex containing the phosphorylation enzyme Tor1, and regulates amino acid metabolism and cell cycle progression. The results of previous studies with *met30* mutants and TORC1 inhibitors have suggested that TORC1 is directly or indirectly involved in the activation of SCF-Met30 [37]. The F-box is known to recognize phosphorylated proteins [38]. Hence, TORC1 potentially phosphorylates unknown proteins related to proline utilization, leading to the recognition and ubiquitination of unknown substrates by SCF-Met30. Future experiments involving gene disruptions related to TORC1 and observation of the SCF-Met30 dissociation states will be needed.

## 4. Conclusions

Wine, a traditional alcoholic beverage, is typically produced using wine yeast, most commonly *S. cerevisiae*. In the highly competitive wine industry, product innovation is essential for maintaining an edge, making the development of novel wine yeast strains a key focus to meet the growing demand for high-quality wines. Proline, a major amino acid present in grape musts, has concentrations ranging from 2.5 mM to 10 mM [4,39,40,41]. However, proline is minimally utilized by yeast during winemaking. This limited utilization negatively impacts both wine quality and production efficiency, leading to proline being labeled “the most wasteful nitrogen source”. Despite having been recognized as an issue for over 50 years, the mechanisms behind yeast’s low consumption of proline during fermentation have remained elusive [2]. Our present data suggest that Can1 and Met30 control proline utilization through independent pathways. Previous studies have shown that Put4 endocytosis is a key event in the inhibition of proline utilization [14,42]. Therefore, our hypothesis is that Met30 and Can1 independently induce Put4 endocytosis, leading to the inhibition of proline utilization in yeast cells. Furthermore, we found that three factors—(1) ammonium ions, (2) methionine or cysteine, and (3) any amino acids except valine, tryptophan, or leucine—cooperatively participate in the inhibition of proline utilization via the Met30 pathway. This study could hold promise for developing wine yeast strains that could efficiently assimilate proline during fermentation.

## Data Availability

The whole-genome sequence and RNAseq data supporting the findings of this study are available from the DDBJ Sequenced Read Archive (https://www.ddbj.nig.ac.jp/index-e.html) under the accession numbers DRR352981, DRR352983, DRR544840, and DRR544841.

## Supplementary Materials

The following supporting information can be downloaded at https://www.mdpi.com/article/10.3390/microorganisms12122510/s1. Table S1: Yeast strains used in this study. Table S2: Oligo DNA used in this study. Figure S1: Regulation of Met4 activity by SCF-Met30. Figure S2: Features of Met30.
