# Supplementary material for: The Yeast F-Box Protein Met30 Regulates Proline Utilization Independently of Transceptor Can1 Under Nutrient-Rich Conditions
Source: Microorganisms. 2024 Dec 5;12(12):2510. doi: 10.3390/microorganisms12122510 (PMC11679997; doi:10.3390/microorganisms12122510)
Supplement: Supplementary file 1 [file microorganisms-12-02510-s001.zip › microorganisms-3322049-supplementary.pdf]

Supplementary materials

The Yeast F-box protein Met30 regulates proline utilization independently of the transceptor Can1 under nutrient-rich conditions

Akira Nishimura[1*], Ryoya Tanahashi[1,2], and Hiroshi Takagi[1**]

[1]*Division for Research Strategy, Institute for Research Initiatives, Nara Institute of Science and Technology, 8916-5 Takayama-cho, Ikoma, Nara, Japan*

[2]*Department of Food Science and Technology, University of California Davis, One Shields Ave, Davis, CA, USA*

[*] Corresponding author. Email: nishimura@bs.naist.jp

[**] Corresponding author. Email: hiro@bs.naist.jp

This PDF file includes:

Tables S1-S2

Figures S1-S2

Table S1 Yeast strains used in this study.

Strain	Genotype	Description
WT	X2180-1A, MATa *SUC2 mal mel gal CUP1*	Wild type strain
pro1Δcar2Δ	*pro1::hphNT1 car2::natNT2*	Proline auxotrophic strain
pro1Δcar2Δcan1Δ	*pro1::hphNT1 car2::natNT2 can1::kanMX4*	Proline auxotrophic strain with *CAN1* deletion
pro1Δcar2Δcan1Δ$met30^{D361G}$	*pro1::hphNT1 car2::natNT2 can1::kanMX4* $met30^{D361G}$	Proline auxotrophic strain with *CAN1* deletion and *MET30 mutation*
pro1Δcar2Δ$met30^{D361G}$	*pro1::hphNT1 car2::natNT2* $met30^{D361G}$	Proline auxotrophic strain with *MET30 mutation*
can1Δ	*can1::kanMX4*	*CAN1* deletion
$met30^{D361G}$	$met30^{D361G}$	*MET30 mutation*
can1Δ$met30^{D361G}$	*can1::kanMX4* $met30^{D361G}$	*CAN1* deletion and *MET30 mutation*

Table S2 Oligo DNA used in this study.

Name	Sequence (5' → 3')	Description
gRNA- MET30 Fw	CGGGTGGCGAATGGGACTTTCGGTTATGTA GTTCCAAACAGTTTTAGAGCTAGAAATAGC	For introduction of *met30*^{D361G} mutation by CRISPR/Cas9 system
gRNA-MET30 Rv	GCTATTTCTAGCTCTAAAACTGTTTGGAAC TACATAACCGAAAGTCCCATTCGCCACCCG	
MET30 A1082G dsDNA Fw	CGTCAAGACATTATATTTTGACGATAGAAA GCTGATTACGGGCTCGCTCGG CAAGACGATTCGTGTTTGGAACTACATAAC CGGTGAATGCATTTCCACGT	
MET30 A1082G dsDNA Rv	ACGTGGAAATGCATTCACCGGTTATGTAGT TCCAAACACGAATCGTCTTGC CGAGCGAGCCCGTAATCAGCTTTCTATCGT CAAAATATAATGTCTTGACG	
SUL1 qPCR Fw	GGGTCATTTTCCAGATCTGCTT	For qPCR
SUL1 qPCR Rv	CGCCAGTGAATACCCCAGAA	
ACT1 qPCR Fw	CACCAACTGGGACGATATGGA	
ACT1 qPCR Rv	GGCAACTCTCAATTCGTTGTAGAA	

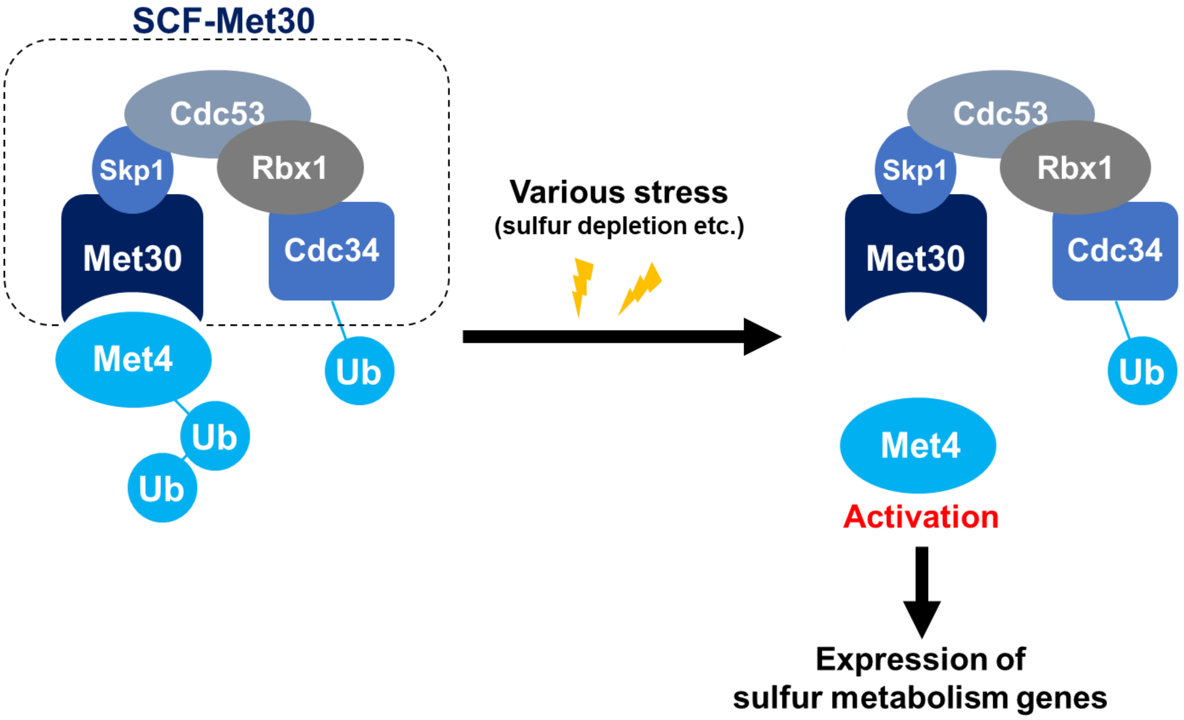

Figure S1 Regulation of Met4 activity by SCF-Met30. Under normal conditions, Met30 binds to the transcription factor Met4, which is involved in sulfur metabolism, ubiquitinating it and thereby suppressing the transcriptional activity of Met4. Conversely, when activation of the sulfur metabolism pathway, such as during sulfur depletion, is necessary, the SCF complex dissociates, leading to deubiquitination of Met4 and rapid transcription of downstream genes controlled by Met4.

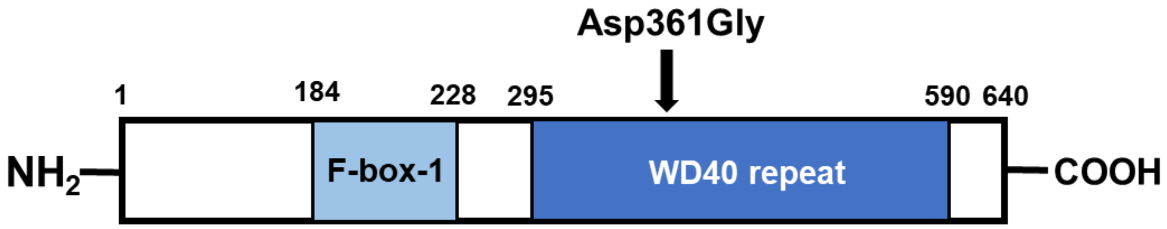


```
                 352      361      370
S. cerevisiae    DRKLITGSLDKTIRVWNYI
S. kluyveri      DQKLITGSLDKTIRVWNYQ
S. bayanus       DRKLITGSLDKTIRVWNYI
S. paradoxus     DRKLITGSLDKTIRVWNYI
S. pombe         QCKLISGSMDKTIRIWNYR
C. glabrata      DQKLITGSLDKTIRVWNYI
C. albicans      NQKLISGGLDSTIKVWNYH
K. lactis        DQKMITASLDKTIRVWNYI
A. gossypii      DQKLITGSLDKTIRVWNYV
D. hansenii      SQKLITGGLDSTIKVWNYH
```

Figure S2 Features of Met30. Upper panel shows multiple domains and a site of amino acid change (Asp361Gly) within Met30 in strains Mutant-1 and Mutant-2. Met30 comprises an F-box-1 domain, which serves as the binding site with the scaffold protein Skp1 of the SCF E3 ubiquitin ligase complex, and a WD40 repeat domain that directly interacts with substrates. Lower panel indicates the sequence alignment of the Met30 proteins from *Saccharomyces cerevisiae* (*S. cerevisiae*), *Saccharomyces kluyveri* (*S. kluyveri*), *Saccharomyces bayanus* (*S. bayanus*), *Saccharomyces paradoxus* (*S. paradoxus*), *Schizosaccharomyces pombe* (*S. pombe*), *Candida glabrata* (*C. glabrata*), *Candida albicans* (*C. albicans*), *Kluyveromyces lactis* (*K. lactis*), *Ashbya gossypii* (*A. gossypii*), and *Debaryomyces hansenii* (*D. hansenii*). Residues are numbered according to the *S. cerevisiae* Met30 and Asp361 is highlighted in a black box.
